# Peptide Extract from* Olivancillaria hiatula* Exhibits Broad-Spectrum Antibacterial Activity

**DOI:** 10.1155/2018/6010572

**Published:** 2018-12-23

**Authors:** Edward Ntim Gasu, Hubert Senanu Ahor, Lawrence Sheringham Borquaye

**Affiliations:** ^1^Central Laboratory, Kwame Nkrumah University of Science and Technology, Kumasi, Ghana; ^2^Department of Chemistry, Kwame Nkrumah University of Science and Technology, Kumasi, Ghana

## Abstract

Increasing reports of infectious diseases worldwide have become a global concern in recent times. Depleted antibiotic pipelines, rapid and complex cases of antimicrobial resistance, and emergence and re-emergence of infectious disease have necessitated an urgent need for the development of new antimicrobial therapeutics, preferably with novel modes of action. Due to their distinct mode of action, antimicrobial peptides offer an interesting alternative to conventional antibiotics to deal with the problems enumerated. In this study, the antimicrobial potential of the peptide extract from the marine mollusc,* Olivancillaria hiatula, *was evaluated* in vitro.* Agar diffusion and broth dilution techniques were used to evaluate microbial susceptibility to the peptide extract. Microplate-based assays were also used to investigate time-dependent growth inhibition profiles of microbes in the presence of peptide and evaluate the peptide's ability to modulate the activities of standard antibiotics. Both Gram-positive and Gram-negative bacteria were inhibited by the peptide extract in the agar diffusion assay. The minimum inhibitory concentration (MIC) of peptide against test microorganisms was between 0.039 and 2.5 mg/mL. At the MIC, the peptide extract was bacteriostatic towards all tested microorganisms but bactericidal to* Staphylococcus aureus. *In the presence of the peptide extract, a prolonged lag phase was observed for all microbes, similar to standard ciprofloxacin. When administered together, peptide extracts enhanced the activities of ciprofloxacin and cefotaxime and were antagonistic towards erythromycin but indifferent towards metronidazole. Taken together, these results show the broad-spectrum antibacterial activity of peptide extract from* Olivancillaria hiatula *and demonstrate that antimicrobial peptides can be employed in combination with some conventional antibiotics for improved effects.

## 1. Introduction

At the beginning of the 20^th^ century, infectious diseases were reported to be the leading cause of global morbidity and mortality. The discovery of the penicillins and other antibiotics improved this grim outlook a bit, with increased optimism that the war against infectious diseases was under control [1, 2]. Between 1930 and 2000, there was a tremendous supply of antibiotics and arsenals of other antimicrobials for clinical and veterinary use. Antimicrobials such as penicillins, tetracyclines, macrolides, cephalosporins, quinolones, aminoglycosides, oxazolidines, and glycopeptides revolutionized the field of medicine and increased life expectancy remarkably [3, 4]. Infectious diseases, however, still remain a concern. Globally, they are the second leading cause of deaths and the third leading cause of death in developing countries [5, 6].

In the last few decades, the world has undeniably faced a postantibiotic era characterized by multidrug resistance, where most microbes are escaping the effect of existing antibiotics [7, 8]. To further compound the situation, there is a marked decline in research and development of antimicrobials [7] and this is a major threat to global health. This decline in the antibiotic pipeline, the inevitable development of resistance that follow the introduction of new antibiotics [9] coupled with emergence, and reemergence of infectious diseases have led to a pressing need for new antimicrobial agents to be unearthed to salvage this dire situation.

To overcome the menace of antimicrobial resistance (AMR), various strategies have been proposed. These include combination therapy [10], supplementing antibiotics with adjuvants [11], modifying old antibiotics to improve antimicrobial activity [12], and searching nature for new antimicrobial agents [7]. While combination therapy seems to be at the risk of toxicity and antagonism, modifying old antibiotics could expand the spectrum of resistance acquisition strategies employed by microorganisms and lead to even further complications. The search for novel antimicrobial agents, antibiotics, or lead compounds with unconventional modes of action from nature is potentially a promising route to tackle the problem.

Most antibiotics owe their source to the terrestrial ecosystem: fungi, soil-borne bacteria, and some plants are examples. The aquatic (marine) ecosystem has languished behind the terrestrial ecosystem in the search for remedies with novel mechanisms of action [13]. However, exploring sources such as the marine environment could lead to the discovery of chemical and biological novelties as well [14]. A number of works on several extracts of marine organisms have shown interesting antimicrobial, antioxidant, antimalarial, anti-inflammatory, and anticancer activities. In fact, some metabolites possessing these properties have been isolated and characterized [15]. Antimicrobial peptides from marine invertebrates provide a novel class of compounds possessing remarkable antimicrobial activities as well as slower rates of resistance acquisition by bacteria [16, 17] that could be explored in the quest for new antimicrobial therapeutics.

Antimicrobial peptides (AMPs) are abundant in nature among plants and various animal families. They are mostly cationic and amphipathic. Due to their amphipathicity, they are able to achieve high concentrations in both aqueous environments and within membranes of organisms. AMPs exhibit a broad-spectrum antimicrobial activity since they constitute the first line of defense of both animals and plants against the attack of microbes. Microbial killing is usually as a result of rapid interaction of the AMP with the microbial outer membrane which leads to membrane disruption, release of cytoplasmic constituents, and a halt to cellular activities [18–21]. Little work is ongoing concerning peptides from Ghanaian marine invertebrates, but crude peptides of* Galatea paradoxa* and* Patella rustica* have been reported to possess some antimicrobial activity [22].

*Olivancillaria hiatula *(*O. hiatula*), a marine gastropod belonging to the family Olividae, is ubiquitous on the shores of Eikwe in the Western Region of Ghana.* O. hiatula* is benthic, and its sessile life form makes it prone to harsh environmental conditions and varying microbial attacks. We have recently shown that solvent extracts from the body tissue of* O. hiatula* possess impressive ability to reduce inflammation* in vivo* [23]. We hypothesized its whole-body tissue as a potential source of antimicrobial peptides. The antibacterial activities and antibiotic-modulating effect of peptides extracted from the whole-body tissue of* O. hiatula* were therefore investigated in this study.

The peptide extract from* O. hiatula* was observed to possess broad-spectrum antimicrobial activity against selected human pathogens. Bacterial growth kinetic studies demonstrated a prolonged lag time with a high reduction in bacterial growth within that period in the presence of peptides at subminimum inhibitory concentrations. Generally, bacteriostatic activity was observed for most of the organisms. Modulation studies revealed that the peptides enhanced the activity of ciprofloxacin and cefotaxime, antagonistic towards erythromycin but indifferent towards metronidazole activity.

## 2. Methods

### 2.1. Sample Collection and Identification

Samples were collected by convenience sampling from Eikwe (4° 58 ‘00” N 2° 28 ‘47” W), a town in the Nzema East Municipality of the Western Region of Ghana. They were transported on ice to laboratories in the Department of Chemistry, Kwame Nkrumah University of Science and Technology (KNUST), Kumasi, and stored at 4°C. Organism was identified with help from the Fisheries and Marine Sciences Department of the University of Ghana, Legon. The Global Biodiversity Information Facility (GBIF) database [24] was used to confirm the taxonomy and sample was identified as* O. hiatula*.

### 2.2. Peptide Extraction

The shells of the molluscs were removed and the whole-body tissues washed and blended. Hundred grams of the blended body tissue was homogenized with 60 mL of 10% (v/v) acetic acid and kept for 12 hours at 4°C. The extract obtained was centrifuged at 5000 rpm (SciSpin ONE, UK) for 10 minutes and the supernatant decanted. Ice-cold acetone (25 mL) was then added to the supernatant and kept at 4°C for 24 hours to precipitate peptides. The precipitates were collected by centrifuging at 5000 rpm for 15 minutes and discarding the supernatant. Precipitates were then frozen at -80°C. Nitrogen gas was used to blow out traces of solvents after freezing at -80°C. The peptides were reconstituted in 25% acetonitrile (ACN) prepared in 0.1% trifluoro acetic acid (TFA) to give 20 mg/mL stock solution [28] and stored at 4°C prior to use.

### 2.3. Characterization by Infrared Spectroscopy

The infrared spectrum of the peptide was determined using a Fourier Transform infrared (FTIR) equipment (UATR Two, PerkinElmer). The regions between 4000 cm^−1^ and 400 cm^−1^ were scanned. This was then followed by baseline correction. Dried extract obtained from lyophilization was used.

### 2.4. Antimicrobial Assays

#### 2.4.1. Microbial Cultures

In this study, nine test bacterial strains (2 Gram-positives and 7 Gram-negatives) were used to assess the antimicrobial properties of the extracts. The Gram-positive bacteria used were* Staphylococcus aureus* ATCC 25923 (*S. aureus*) and* Enterococcus faecalis* ATCC 29212 (*E. faecalis*). Gram-negative bacteria included* Escherichia coli* ATCC 25922 (*E. coli*),* Proteus mirabilis* ATCC 4175 (*P. mirabilis*),* Pseudomonas aeruginosa* ATCC 4853 (*P. aeruginosa*), and clinical strains of* Klebsiella pneumonia *(*K. pneumonia)*,* Salmonella paratyphi* (*S. paratyphi)*,* Neisseria gonorrhea* (*N. gonorrhea), *and* Vibrio cholera *(*V. cholera)*. All microbial strains were obtained from the Department of Pharmaceutical Microbiology, Faculty of Pharmacy and Pharmaceutical Sciences of the College of Health Science, KNUST.

#### 2.4.2. Inoculum Preparation

Bacterial isolates were streaked onto nutrient agar (Oxoid, United Kingdom) plates and incubated for 18–24 hours at 37°C. Using the direct colony suspension method, suspensions of the organisms were made in nutrient broth and incubated overnight at 37°C. These overnight cultures were used for the determination of antimicrobial activity using the well diffusion assay. For the remaining tests, colony suspensions in sterile saline was adjusted to 0.5 McFarland standard and further diluted in sterile double strength nutrient broth (~2 × 10^5^ CFU/mL) [29].

### 2.5. Agar Well Diffusion Assay

Twenty-five milliliters of freshly prepared sterile nutrient agar (cooled to 40–50°C) was poured into sterile Petri dishes containing 10 *μ*L of overnight cultures and swirled to ensure a homogenous spread of the organisms. This was allowed to solidify. Three equidistant wells of 6 mm in both diameter and depth were made on the plates using sterile cork borers. 100 *μ*L of prepared peptide solution was then dispensed into the wells, allowed to equilibrate at room temperature for 30 minutes, and then incubated overnight at 37°C. Zones of growth inhibition (in mm) were measured as the diameter of the clear zone around each well. The assay was performed in independent triplicates and the averages of the three experiments taken. Ciprofloxacin (Sigma Aldrich, Michigan, USA) was used as reference antimicrobial agent (positive control) for bacteria strains while 25 % ACN in 0.1 % TFA was used as negative control [28].

### 2.6. Minimum Inhibitory Concentration

Minimum Inhibitory Concentration (MIC) of the peptide extract was determined by the broth microdilution method described by Wiegand [29]. Ten to twenty-four serial twofold dilutions of peptide or standard antibiotic (Ciprofloxacin) were prepared to obtain a final concentration range of 2.5 to 4.88 × 10^−3^ mg/mL and 500 to 5.96 × 10^−5^*μ*g/mL for peptide and ciprofloxacin, respectively, in a microtiter plate. Fifty microliters of double strength nutrient broth containing an inoculum size of ~2.0 × 10^5^ CFU/mL was added to each well. The total volume of each well was 100*μ*L. The plates were covered and incubated at 37°C for 24 hours. Twenty microliters of 1.25 mg/mL 3-(4, 5-dimethylthiazol-2-yl)-2,5-diphenyltetrazolium bromide (MTT) was added to each well and incubated for 30 minutes at 37°C. The MIC was determined as the lowest concentration of peptide extract or drug that inhibited growth of test organism. This was indicated by the absence of purple coloration upon the addition of the MTT dye and incubation. All tests were performed in triplicate.

### 2.7. Minimum Bactericidal Concentration (MBC)

Minimum bactericidal concentrations (MBC) of the peptide extracts were determined by the same procedure as the MIC assay. After the 24-hour incubation period, 50 *μ*L aliquots from wells with peptide concentrations greater than the MIC were plated on sterile agar plates. Agar plates were incubated at 37°C for 24 hours. MBC was recorded as the lowest extract concentration killing 99.9 % of the bacterial, i.e., least peptide concentration that showed no visible growth of the microorganisms on the surface of the nutrient agar. Each experiment was repeated three times.

### 2.8. Evaluation of Bactericidal and Bacteriostatic Capacity of Peptide Extract

The ratio of MBC/MIC was used to characterize the antimicrobial activity of peptide extracts. When the ratio of MBC/MIC ≤ 2, the effect was considered as bactericidal and a ratio ≥4 defined as bacteriostatic [25].

### 2.9. Microplate-Based Turbidimetric Growth Inhibition Assay

Growth inhibition of test organisms in the presence of peptide was studied using the microplate inhibition assay [30, 31] with slight modifications. In this assay, peptide extract was serially diluted from 4× MIC concentration through to 0.25× MIC peptide concentration for each organism after which 50 *μ*L of nutrient broth containing a microbial inoculum size of ~2.0 × 10^5^ CFU/mL was added. Microplates were incubated at 37°C and optical density at 600 nm (OD_600_) determined at 2 hourly intervals with a microplate reader (Synergy H1 multimode plate reader, Germany). The OD_600_ values obtained were plotted against time and were used to illustrate the inhibitory activity of the peptide of* O. hiatula* against the various test organisms.

### 2.10. Modulation Studies

The ability of peptide extracts at sub-MIC concentrations to modulate the activity of standard antibiotics was evaluated. In this experiment, the MIC of standard antibiotics against the microbes and the MIC of the antibiotics in the presence of sub-MIC concentration of the peptide were determined. The microbial resistance modulation tests were performed according to a modified procedure described by Wiegand and coworkers [29]. Twenty-four serial twofold dilutions of standard antibiotics; Ciprofloxacin (Sigma Aldrich), Metronidazole (Sigma Aldrich), Erythromycin (Alfa Aesar), and cefotaxime (Alfa Aesar) were prepared to obtain final concentration ranges of 500 to 5.96 ×10^−5^*μ*g/mL. Fifty microliters of nutrient broth containing a microbial inoculum size of ~2.0 × 10^5^ CFU/mL was added to each well. The reference antibiotics were tested against all microorganisms. MICs were determined after incubation of plates for 24 hours and upon the addition MTT to the medium in the wells.

Subinhibitory concentrations of 20 *μ*g/mL of the peptide solution and various dilutions of standard antibiotics plus the same inoculum size were mixed and then incubated overnight at 37°C. MICs of antibiotics in the presence of the peptides were determined as described earlier. All tests were performed in triplicate.

Modulation factor (MF) was calculated and used to evaluate the antimicrobial effects of the peptide extract on the MIC of various antibiotic used.

(1) $$\begin{array}{c} MF=\frac{MIC\;\left(antibiotic\right)}{MIC\;\left(antibiotic+modulator\right)}. \end{array}$$

A modulation factor >2 was set as the cut-off for biologically significant modulation [32].

The change in MIC was computed using [33]

(2) Change  in  MIC=MIC  Antibiotic−MIC  Antibiotic+PeptideMIC  Antibiotic×100.

### 2.11. Data Analyses

GraphPad Prism Version 6.0 for Windows (GraphPad Software, San Diego, CA, USA) and Microsoft Excel 2007 were used for all data analyses and graphs.

## 3. Results

### 3.1. Infrared Characterization

**Figure 1:**
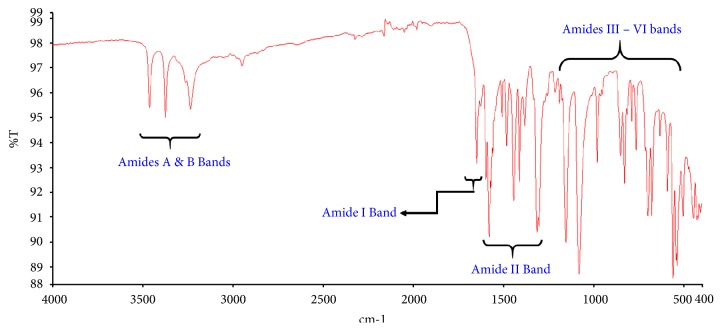
Fourier Transform infrared (FTIR) spectrum of peptide extract of* Olivancillaria hiatula*. Amides A&B bands spans 3100–3500 cm^−1^, Amide I band is from 1600 to 1700cm^−1^, Amide II band is from 1480 to 1600 cm^−1^, and the region from 500 to 1300 cm^−1^ represents Amides III–VI bands [26, 27].

The spectrum obtained from the FTIR showed prominent peaks of a typical peptide. Prominent peaks consistent with stretching and bending vibrations of N-H, C=O and C-H were observed (Figure 1).

### 3.2. Antimicrobial Assay

The peptide extract showed a broad-spectrum antimicrobial activity with impressive activities against all microorganisms. All extracts were tested at a concentration of 5 mg/mL for the agar diffusion assay. The highest zone of inhibition (ZI) was recorded against* N. gonorrhea *while no zone of clearance was observed against* V. cholera, S. paratyphi,* and* E. faecalis *at this concentration. When the concentrations were increased between 10 and 50 mg/mL, however, clear zones of inhibition were observed for those 3 microorganisms (Table 1).

### 3.3. Minimum Inhibitory Concentration (MIC) of Extracts

Peptide extract from* O. hiatula *demonstrated really good antimicrobial activity with very low MICs recorded. MICs ranged from 2.5 to 0.039 mg/mL against all test organisms. Gram-positive organisms recorded a relatively high MIC of 2.5 mg/mL while the Gram-negative bacteria, especially, recorded much lower MICs (Table 2).* P. mirabilis *and* P. aeruginosa *in particular had very low MICs (39 *μ*g/mL) as can be seen in Table 2.

### 3.4. Minimum Bactericidal Concentration (MBC)

The MBC and the ratio of MBC to MIC were determined for the peptide extract against all test organisms. This ratio indicated the microbiostatic or microbicidal nature of the peptide extract against the test organisms. The lowest MBC (1.25 mg/mL) was recorded for* P. aeruginosa *while relatively higher MBC (≥ 2.5 mg/mL) of peptide were recorded for the remaining test organisms. From the ratio of MBC to MIC, the peptide was seen to have a microbicidal effect against* S. aureus *and a microbiostatic action against the remaining test organisms (Table 2).

### 3.5. Microplate Turbidimetric Growth Inhibition Assay

**Figure 2:**
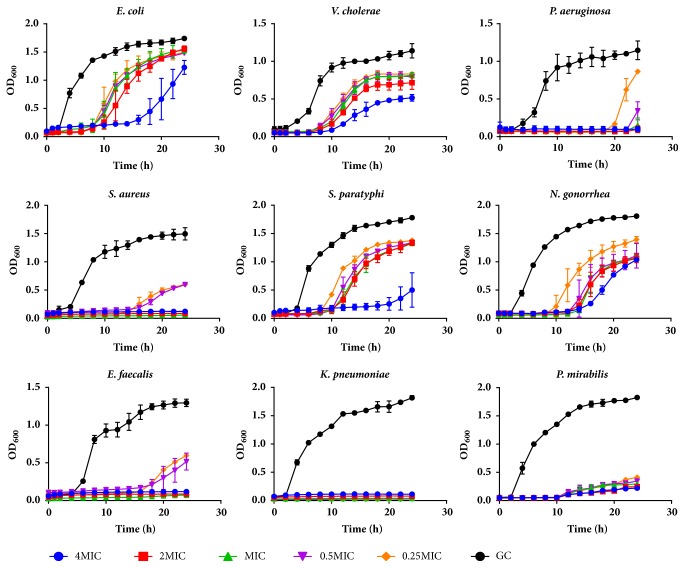
Growth curves of test microorganisms in the presence of varying concentrations of peptide extract. Each data point is the average of 3 replicate experiments (MIC, minimum inhibitory concentration; GC, growth control).

**Figure 3:**
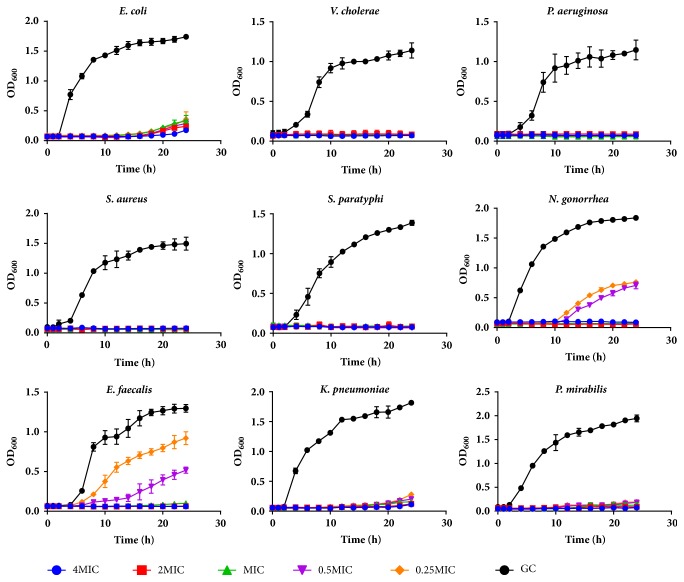
Growth curves of test microorganisms in the presence of varying concentrations of standard ciprofloxacin drug. Each data point is the average of 3 replicate experiments (MIC, minimum inhibitory concentration; GC, growth control).

In the growth inhibition assay of the peptide extract against the test organisms, the growth curves of the test organisms in the presence of 4× MIC, 2× MIC, MIC, 0.5× MIC, and 0.25× MIC of the peptide extract were reduced comparative to the growth curves of the control (test organism in the absence of peptide). The lag phases of the test organisms were prolonged for an average of 16 hours while the log phases were also reduced in the presence of the peptide. Growth curves of most test organisms flattened during the 24-hour incubation period in the presence of 2× MIC and 4× MIC of peptide concentration while this effect was observed at even the MIC of ciprofloxacin (Figures 2 and 3). The effects of the peptide extract in inhibiting the growth of the test organism were observed to be concentration dependent (Figure 2).

### 3.6. Antibiotic Modulation

Peptide extract of* O. hiatula* at sub-MIC concentration of 20 *μ*g/mL had noticeable effects on the response of test organism to antibiotics with modulation factor ranging from <0.25 to 524288 (Tables 3–6). When 20 *μ*g/mL of peptide extract was added to varying concentrations of ciprofloxacin and test organisms, the MIC of ciprofloxacin reduced markedly for all test organisms by a factor as high as about 16, 000 (Table 3). Sub-MIC concentration of peptide extract also modulated the action of cefotaxime positively against test organisms (Table 4). There was a reduction in the MIC of cefotaxime in the presence of the peptide extract for all test organisms except* S. aureus and N. gonorrhea* where an increase in MIC was observed. The MIC of* N. gonorrhea* actually doubled under the experimental conditions (Table 4).

The peptide extract did not have any noticeable effect on metronidazole (Table 6) but was antagonistic to erythromycin (Table 5).

## 4. Discussions

Various methods exist for the isolation of peptides from marine invertebrates. In this work, we have utilized the whole-body tissue of* O. hiatula *as our source of antimicrobial peptides. Ice-cold acetone precipitation of peptides from whole-body tissue homogenate afforded crude peptides in appreciable quantities.

The FTIR spectrum of the extract obtained was consistent with reported vibrational spectra of peptides [26, 27, 34]. Amide I band, which is a direct consequence of the carbonyl (C=O) stretching vibrations, was observed at about 1650 cm^−1^. N-H bending and C-N stretching vibrations are the major contributors to Amide II bands and are usually observed from 1480 to 1575 cm^−1^. In this spectrum, Amide II bands turned out to be more prominent with strong absorptions recorded in this region. Amides A and B bands can be observed between 3200 and 3500 cm^−1^. These are usually due to N-H stretching vibrations. Peaks corresponding to Amides III–VI regions (500–1300 cm^−1^) can also be seen in the spectrum. Together, these peaks are indicative of a sample predominantly made up of peptides. IR spectra can be used to estimate secondary structural elements. It is difficult to make any such deductions from the spectrum of this extract since it is presumably a mixture and could contain a number of different peptides. However, the absence of strong Amide I absorptions is conspicuous. Based on this observation, it could be speculated that the extract is rich in *α*-helical peptides [26, 34].

Antimicrobial peptides (AMPs) usually exhibit broad-spectrum antimicrobial activity and have been suggested as an alternative to counter the menace of antimicrobial resistance. Because AMPs are usually membrane targeting, microbial resistance would probably involve the architectural redesigning and/or compositional variation of the entire cell lipid membrane of the microorganism [21]. Such a venture would most likely be very costly and difficult to achieve for microorganisms. AMPs therefore represent a viable therapeutic option.

Peptide extract from* O. hiatula *was active against both Gram-positive and Gram-negative bacteria. Microbial susceptibility was evaluated using the agar well diffusion and broth microdilution methods. Even though some microbes (*S. paratyphi, V. cholera, *and* E. faecalis*) required much higher peptide concentration for activity to be observed in the agar diffusion assay, they showed really good activities in the broth microdilution test. The broth microdilution assay is regarded as being more sensitive relative to the agar diffusion assay for screening antimicrobial natural products [35]. Properties of the natural product such as pH, solubility, volatility and diffusion in agar all influence results of the agar diffusion assay but not broth microdilution assay [36, 37]. The low MICs recorded against* N. gonorrhea* and* P. aeruginosa* is impressive and hence extract is considered to be very active [37]. In general, the MIC values recorded are much lower than those recorded for peptide extracts from* Patella rustica* and* Galatea paradoxa*[22] as well as methanol and ethyl acetate extracts of* Littorina littorea* and* Galatea paradoxa* [38]. These MICs, however, are in the range of those recorded for the antimicrobial peptide pexiganan, an antimicrobial peptide that has advanced furthest in clinical trials for the treatment of diabetic foot ulcers. MICs for pexiganan ranged from 16 to 32 *μ*g/mL [39, 40]. There is a strong positive correlation between *α*-helical content and antimicrobial activity [41, 42]. The impressive activities recorded against both Gram-positive and Gram-negative bacteria supports the notion that the major secondary structural elements in* O. hiatula *peptide extract are *α*-helices, which was speculated from the IR data.

To investigate the kind of inhibitory effects that the peptide extract had on the various bacteria studied, the minimum bactericidal concentration (MBC), defined as the lowest extract concentration killing 99.9 % of the bacterial inocula after 24-hour incubation at 37°C, was recorded. At the MIC, a bacteriostatic effect was observed for all bacteria, except* S. aureus* where a bactericidal effect was observed. Above the MIC, peptide extract was found to possess a bactericidal effect. The activity of most AMPs is concentration dependent. An increase in peptide: lipid ratio across the membrane of microorganism greatly enhances the peptide's ability to penetrate and disrupt membrane integrity. Ion channel formation, transmembrane pore formation, and membrane rupture which all result in microbial death are more prevalent at higher peptide concentrations [43]. This effect can be observed clearly in the growth curves of the various bacteria in the presence of varying peptide concentrations where a prolonged lag phase is recorded at 2× - and 4× MIC. The growth curves of* S. aureus, S. paratyphi, P. mirabilis, *and* P. aeruginosa *and to a lesser extent* E. faecalis *in the presence of peptides (Figure 2) were similar in shape to that of the standard drug, ciprofloxacin (Figure 3).

While therapeutic agents can be used in isolation to elicit specific effect(s), combination therapy is fast becoming the norm due to several advantages associated with it. Combination therapy could possibly reduce emergence of drug resistant microbes as the microorganism has to adapt to two or more drugs with different* modus operandi*. Toxicity associated with high doses could also be eliminated in combination therapy since lower doses of the drugs will be required to achieve comparable levels of efficacy in single drug therapy. Finally, the range of pathogens that could be targeted may be expanded depending on the individual drugs present in that particular combination [44]. Identification of AMPs that can be combined with orthodox antibiotics to be used for the treatment of infections has a good potential to expand available therapeutic options.

To evaluate the possible effect of the peptide extract of* O. hiatula *on some standard antibiotics, modulation experiments were set up. Subinhibitory concentration of peptide extract remarkably decreased the MICs of ciprofloxacin against all test microorganisms. When peptide was combined with cefotaxime, the MICs against almost all test microorganisms were also reduced. For erythromycin and metronidazole, a different trend was observed, with higher MICs being recorded for erythromycin and no appreciable change observed in the case of metronidazole. Both sets of antagonistic and synergistic effects of antimicrobial peptides in combination with antibiotics have been reported in literature [45, 46]. The synergistic interaction between peptides and antibiotics could be a result of the membrane permeability action of peptides or pore formation in the bacterial membrane. This leads to disruption of membrane integrity and easy penetration of antibiotics into bacterial cells where they cause greater damage [39, 40, 46]. The antimicrobial peptides, WR12 and D-IK8, have been shown to possess potent synergism with most topical antibiotics (fusidic acid and mupirocin) and systemic antibiotics (daptomycin, teicoplanin, vancomycin, linezolid, ciprofloxacin, meropenem, and oxacillin) [46]. Short peptide chains are known to confer bacterial resistance towards some macrolide antibiotics, especially erythromycin. Macrolide resistance occurs via modification of the drug binding site (either via allosteric mutations of direct mutations of amino acid residues in the vicinity of the binding pocket) [45, 47], action of specialized antibiotic efflux pumps [48], and the action of short peptides [45, 49]. Short peptides bind to the macrolide and form an inactive complex or act directly on the ribosome to inhibit or terminate translation [45].

## 5. Conclusions

The broad-spectrum antibacterial activity of the peptide extract of* O. hiatula *has demonstrated this study. Peptide extract was shown to be bacteriostatic at the MIC but bactericidal at twice and quadruple MICs. In the presence of the peptide extract, a prolonged lag phase was observed in the growth patterns of all test microorganisms. The peptide extract was also found to be synergistic when used with ciprofloxacin and cefotaxime but antagonistic towards erythromycin and indifferent to metronidazole. Together, these results demonstrate the utility of peptide extracts from* O. hiatula *as potential source of potent antimicrobial agents. Efforts to isolate and characterize the antimicrobial peptides in the extract mix are currently underway in our laboratories.

## Figures and Tables

**Table 1:** Zones of inhibition (mm) of peptide extract against test microorganisms.

		Zone of inhibition (mm)	
Microorganism (Gram status)	Peptide Extract (5 mg/ml)	Positive control Cipro (1mg/ml)	Negative control
*E. coli *(-)	30.9 ± 0.2	51.5 ± 1.3	-
*K. pneumonia *(-)	28.9 ± 0.8	48.8 ± 1.2	-
*S. paratyphi *(-)	27.4 ± 0.5^**#**^	25.0 ± 6.1	-
*P. mirabilis *(-)	12.1 ± 0.6	41.0 ± 0.2	-
*N. gonorrhea *(-)	31.0 ± 0.4	40.0 ± 0.8	-
*V. cholera *(-)	30.0 ± 0.7**∗**	40.3 ± 0.6	-
*P. aeruginosa *(-)	27.0 ± 0.7	53.3 ± 0.9	-
*S. aureus *(+)	28.5 ± 2.1	39.2 ± 0.9	-
*E. faecalis *(+)	33.2 ± 3.0^**#**^	46.5 ± 1.9	-

Values reported as mean ± standard deviation of three replicate experiments; **∗** and **#** activity at 10 mg/mL and 50 mg/mL, respectively (ZI not observed at 5mg/mL); negative control (25 % ACN in 0.1 % TFA).

**Table 2:** MIC, MBC, bacteriostatic and bactericidal effects of peptide extract.

Microorganism	MIC (mg/mL)	MBC (mg/mL)	MBC/MIC	Effect
*E. coli (-)*	0.625	>2.5	>4	static
*K. pneumonia (-)*	1.25	>2.5	>2	static
*S. paratyphi (-)*	0.625	2.5	4	static
*P. mirabilis (-)*	0.039	>2.5	>4	static
*N. gonorrhea (-)*	0.156	2.5	>4	static
*V. cholera (-)*	0.315	2.5	>4	static
*P. aeruginosa (-)*	0.039	1.25	>4	static
*S. aureus (+)*	2.5	2.5	1	cidal
*E. faecalis (+)*	2.5	>2.5	>1	Cidal / static

MIC and MBC experiments were replicated thrice; MIC, minimum inhibitory concentration; MBC, minimum bactericidal concentration; MBC/MIC ≤ 2 implies bactericidal; MBC/MIC ≥ 4 implies bacteriostatic [25].

**Table 3:** Co-modulation studies: MICs of ciprofloxacin plus 20 *μ*g/mL of peptide extract.

Organism	MIC (*μ*g/mL)		MF	Change in MIC (%)
	Cip	Cip + P		
*E. coli (-)*	1.95	0.00095	2053	99.95^*** R***^
*K. pneumonia (-)*	0.00095	0.00012	8	87.50^***R***^
*S. paratyphi (-)*	125.00	0.00763	16383	99.99^***R***^
*P. mirabilis (-)*	0.00048	0.00003	16	50.00^*** R***^
*N. gonorrhea (-)*	125.00	62.5	2	50.00^***R***^
*V. cholera (-)*	3.91	0.00095	4116	99.98^***R***^
*P. aeruginosa (-)*	0.24	0.00191	126	99.99^***R***^
*S. aureus (+)*	15.63	1.95	8	87.50^***R***^
*E. faecalis (+)*	0.24	0.0038	64	98.44^*** R***^

MIC experiments were replicated thrice; change in MIC computed using equation (2).

MF: modulation factor, Cip: ciprofloxacin, P: peptide extract, and *R*: reduction in MIC

**Table 4:** Co-modulation studies: MICs of Cefotaxime plus 20 *μ*g/mL of peptide extract.

Organism	MIC (*μ*g/mL)			
	Cef	Cef + P	MF	Change in MIC (%)
*E. coli (-)*	31.25	1.95	16	93.75^*R*^
*K. pneumonia (-)*	31.25	3.91	8	87.50^*R*^
*S. paratyphi (-)*	62.5	3.91	16	93.75^*R*^
*P. mirabilis (-)*	31.25	0.24	130	99.22^*R*^
*N. gonorrhea (-)*	31.25	62.50	0.5	100.00^*I*^
*V. cholera (-)*	62.5	1.19 × 10^−4^	525210	99.99^*R*^
*P. aeruginosa (-)*	31.25	1.19× 10^−4^	262605	100.00^*R*^
*S. aureus (+)*	250.00	>250.00	<1	100.00^*I*^
*E. faecalis (+)*	31.25	1.95	16	93.75^*R*^

MIC experiments were replicated thrice; change in MIC computed using equation (2).

MF: modulation factor, Cef: cefotaxime, P: peptide extract, and R: reduction in MIC, *I*: increase in MIC.

**Table 5:** Co-modulation studies: MICs of Erythromycin plus 20 *μ*g/mL of peptide extract.

Organism	MIC (*μ*g/mL)		MF	Change in MIC (%)
	Eryt	Eryt + CP		
*E. coli*	125	125	1	*N*
*K. pneumonia*	7.8	>7.8	<1	> 100^***I***^
*S. paratyphi*	250	>500	<0.50	> 100^***I***^
*P. mirabilis*	125	250	0.50	100^***I***^
*N. gonorrhea*	500	>500	<1	> 100^***I***^
*V. cholera*	125	>500	<0.25	>100^***I***^
*P. aeruginosa*	125	>500	<0.25	>100^***I***^
*S. aureus*	>500	>500	<1.00	> 100^***I***^
*E. faecalis*	0.24	>7.8	<0.03	>100^***I***^

MIC experiments were replicated thrice; change in MIC computed using equation (2).

MF: modulation factor, Eryt: erythromycin, P: peptide extract, *N*: no change, and *I*: increase in MIC

**Table 6:** Co-modulation studies: MIC values of mtronidazole plus 20 *μ*g/mL of peptide extract.

Organism	MIC (*μ*g/mL)			
	Met	Met + CP	MF	Change in MIC (%)
*E. coli*	>500	>500	<1	> 100^***I***^
*K. pneumonia*	>500	>500	<1	> 100^***I***^
*S. paratyphi*	>500	>500	<1	> 100^***I***^
*P. mirabilis*	>500	>500	<1	> 100^***I***^
*N. gonorrhea*	>500	>500	<1	> 100^***I***^
*V. cholera*	>500	>500	<1	> 100^***I***^
*P. aeruginosa*	>500	>500	<1	> 100^***I***^
*S. aureus*	>500	>500	<1	> 100^***I***^
*E. faecalis*	>500	250	>1	<50^***R***^

MIC experiments were replicated thrice; change in MIC computed using equation (2).

MF: modulation factor, Met: Metronidazole, P: peptide extract, *R:* reduction in mic, and *I*: increase in MIC

## Data Availability

The data used to support the findings of this study are included within the article.
